# Ultrasound-irradiated synthesis of 3-mercaptopropyl trimethoxysilane-modified hydroxyapatite derived from fish-scale residues followed by ultrasound-assisted organic dyes removal

**DOI:** 10.1038/s41598-021-85206-5

**Published:** 2021-03-10

**Authors:** Phitchan Sricharoen, Supalak Kongsri, Chunyapuk Kukusamude, Yonrapach Areerob, Prawit Nuengmatcha, Saksit Chanthai, Nunticha Limchoowong

**Affiliations:** 1 Nuclear Technology Research and Development Center, Thailand Institute of Nuclear Technology (Public Organization), 9/9 Moo 7, Tambon Saimoon, Ongkharak, Nakhon Nayok, 26120 Thailand; 2 Department of Industrial Engineering, Faculty of Engineering, King Mongkut’s Institute of Technology Ladkrabang, Bangkok, 10520 Thailand; 3 Nanomaterials Chemistry Research Unit, Department of Chemistry, Faculty of Science and Technology, Nakhon Si Thammarat Rajabhat University, Nakhon Si Thammarat, 80280 Thailand; 4 Materials Chemistry Research Center, Department of Chemistry and Center of Excellence for Innovation in Chemistry, Faculty of Science, Khon Kaen University, Khon Kaen, 40002 Thailand; 5 Department of Chemistry, Faculty of Science, Srinakharinwirot University, Bangkok, 10110 Thailand

**Keywords:** Pollution remediation, Analytical chemistry, Chemical engineering, Green chemistry, Materials chemistry

## Abstract

We report a novel method for the synthesis of 3-mercaptopropyl trimethoxysilane-modified hydroxyapatite (FHAP-SH) derived from fish-scale residues by using ultrasound irradiation. Scanning electron microscopy, transmission electron microscopy, energy-dispersive spectroscopy, X-ray diffraction, and Fourier transform infrared spectroscopy were used for the FHAP-SH characterization. Then, the organic dye adsorption on the FHAP-SH was monitored through an ultrasound process. After the dye removal optimization, significant improvements were observed in the maximum adsorption capacities for Congo Red (CR, 500 mg g^−1^), Coomassie Brilliant Blue G 250 (CB, 235 mg g^−1^), and Malachite Green (MG, 625 mg g^−1^). The adsorption behaviors of these dyes were fitted by using the Langmuir isotherm model with a high coefficient of determination values ranging from 0.9985 to 0.9969. The adsorption of the three dyes onto FHAP-SH was an endothermic process based on the adsorption thermodynamics model, while the adsorption kinetics analysis of the dyes presented a good alignment with the pseudo-second-order kinetics. The FHAP-SH exhibits a remarkably high adsorption capacity, is inexpensive, and fulfills the ecofriendly requirements of dye wastewater treatment, especially in the textile industry.

## Introduction

Water contamination caused by the use of textile dyes in the fashion manufacturing industry is emerging as a primary source of wastewater. This anthropogenically contaminated water directly enters irrigation and drinking water systems; thus, causing permanent damage to the environment and human health. Dyeing wastewater is hard to treat, and adsorption could be considered a good treatment choice. Our concept of research deals with the recycling of food waste discarded by each household, which can be broken down and reused to prepare new things. In particular, the fish-scale residues that are rich in Ca and P can be recycled and processed into hydroxyapatite (HAP) nanoparticles, a mineral form of the Ca_10_(OH)_2_(PO_4_)_6,_ which is a major part of the bone and teeth mineral^1^. The important characteristics of hydroxyapatite nanoparticles are their capacities of accepting a large amount of cationic/anionic substituents that facilitate their utilization as an adsorbent for the decontamination of toxic pollutants, including the dyes such as Acid Yellow 220^2^, Methylene Blue^3^, Methyl Orange^4^, etc., which may have harmful consequences related to human wellbeing. Although nano-hydroxyapatite has attracted considerable attention in the elimination of poisonous dyes, its surface modification is quite necessary for improving the effectiveness of dye removal. In general, the adsorption capacity of HAP toward target analytes can be increased by the introduction of some chemical functionality/modification into the hydroxyapatite, such as magnetic^5^, Ag, and multiwall carbon nanotube^6^, amino group^7^, and chitosan^8^. Therefore, the novel surface modification approach would be effective in improving the dye removal efficiency of HAP. In this study, 3-mercaptopropyl-trimethoxysilane (MPTS), a thiol group (–SH), was incorporated into hydroxyapatite via condensation with the hydrolyzed Si–OH of MPTMS. The abundance of the–SH ligand in the modified surface can make it a potential material for use as an excellent sorbent. This unique characteristic reveals that the thiol surface has good application potential in pollutant determination and removal from wastewater and other samples^9–13^. HAP and modified HAP have been used in several forms, and several methods, such as hydrothermal reactions^14^, sol–gel synthesis^15^, microwave method^16^, and ultrasound chemical method^17–20^, were used for their synthesis. In the ultrasound technique, after the mixing of the Ca and PO_4_^3−^ solution (from the chemical or biological source) under basic conditions through ultrasound treatment, hydroxyapatite or its modification nanoparticles were obtained by centrifugation followed by drying.

This work reports an ultrasound-irradiated synthesis of hydroxyapatite from fish scales (FHAP), and the surface modification of the synthesized HAP to produce 3-mercaptopropyl-trimethoxysilane (FHAP-SH), which was then used for the removal of Congo Red (CR), Coomassie Brilliant Blue G 250 (CB), and Malachite Green (MG) dyes from water samples by adsorption, followed by a study of their adsorption behaviors. The FHAP-SH sorbents are cheap, available in nature, and do not produce poisonous, dangerous waste. The characterization of the obtained material can be carried out by XRD, FTIR, EDX, SEM, and TEM. Then, the analyses of adsorption optimization, isotherm, thermodynamics, and kinetics were performed in detail. To the best of our knowledge, this work is the pioneer in demonstrating the removal of the three dyes using the novel FHAP-SH combined with ultrasound energy and exhibiting satisfactory results.

## Experimental sections

### Materials and reagents

Congo Red was purchased from Merck (Germany), Coomassie Brilliant Blue G 250 was purchased from Fluka (Switzerland), and Malachite Green oxalate salt and 3-Mercaptopropyl-trimethoxysilane were purchased from Sigma-Aldrich (New Zealand). Hydrochloric acid, sodium hydroxide, and toluene were purchased from QReC (New Zealand). A water purification system (Molsheim, France) was used to produce deionized water with a resistivity of 18.2 Ω cm. All materials and reagents were of analytical grade.

### Ultrasound-irradiated synthesis of FHAP-SH

For FHAP, dehydrated fish scales (5 g) placed in a beaker were mixed with 100 mL of HCl (0.8 M). Ultrasonic power of 0.4 kW, the temperature of 60 °C, and extraction time of 45 min were the conditions used for ultrasonic extraction (40 kHz bath, model VGT-2300B, brand GT SONIC, China). After centrifugation, the liquid phase was collected and NaOH (pH 12) was added dropwise. Then, the mixture was ultrasonicated (0.4 kW, room temperature) for 30 min, followed by washing the particles with deionized water, oven drying, and finally grinding to obtain the powder of FHAP. For FHAP-SH, 1 g of FHAP and 1 mL of 3-mercaptopropyl-trimethoxysilane were dispersed in 20 mL of toluene and then sonicated under a power of 0.4 kW for 1 h. Afterward, the sample was centrifuged and dried to obtain a white powder of FHAP-SH.

### Ultrasound-assisted organic dye removal

For the adsorption experiment, 0.02 g of adsorbent and 2% w/v of NaCl in 10 mL of the dye solution (pH 6) were sonicated at 0.4 kW and 45 °C for 60 min for CR and MG or 90 min for CB. Thereafter, the dye sorbent was centrifuged, and the supernatant was examined using a UV–visible spectrophotometer at 495, 595, and 620 nm for CR, CB, and MG, respectively. All data were expressed as the mean ± SD of triplicate determinations.

## Results and discussion

### FHAP-SH characterization

**Figure 1:**
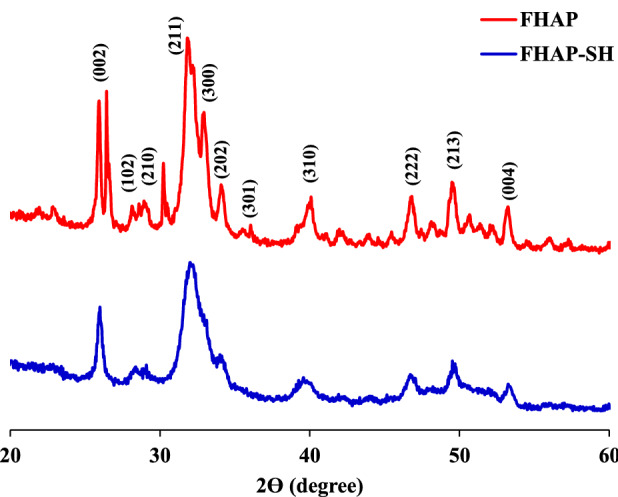
XRD patterns of the FHAP and FHAP-SH.

**Figure 2:**
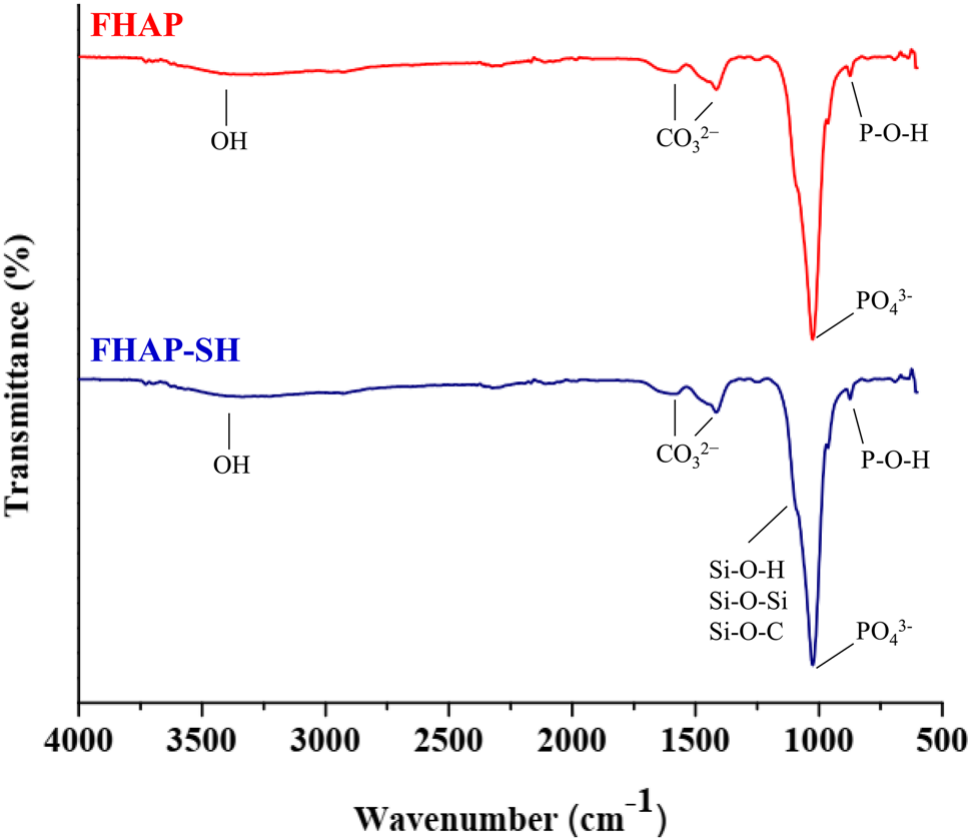
FTIR spectra of the FHAP and FHAP-SH.

**Figure 3:**
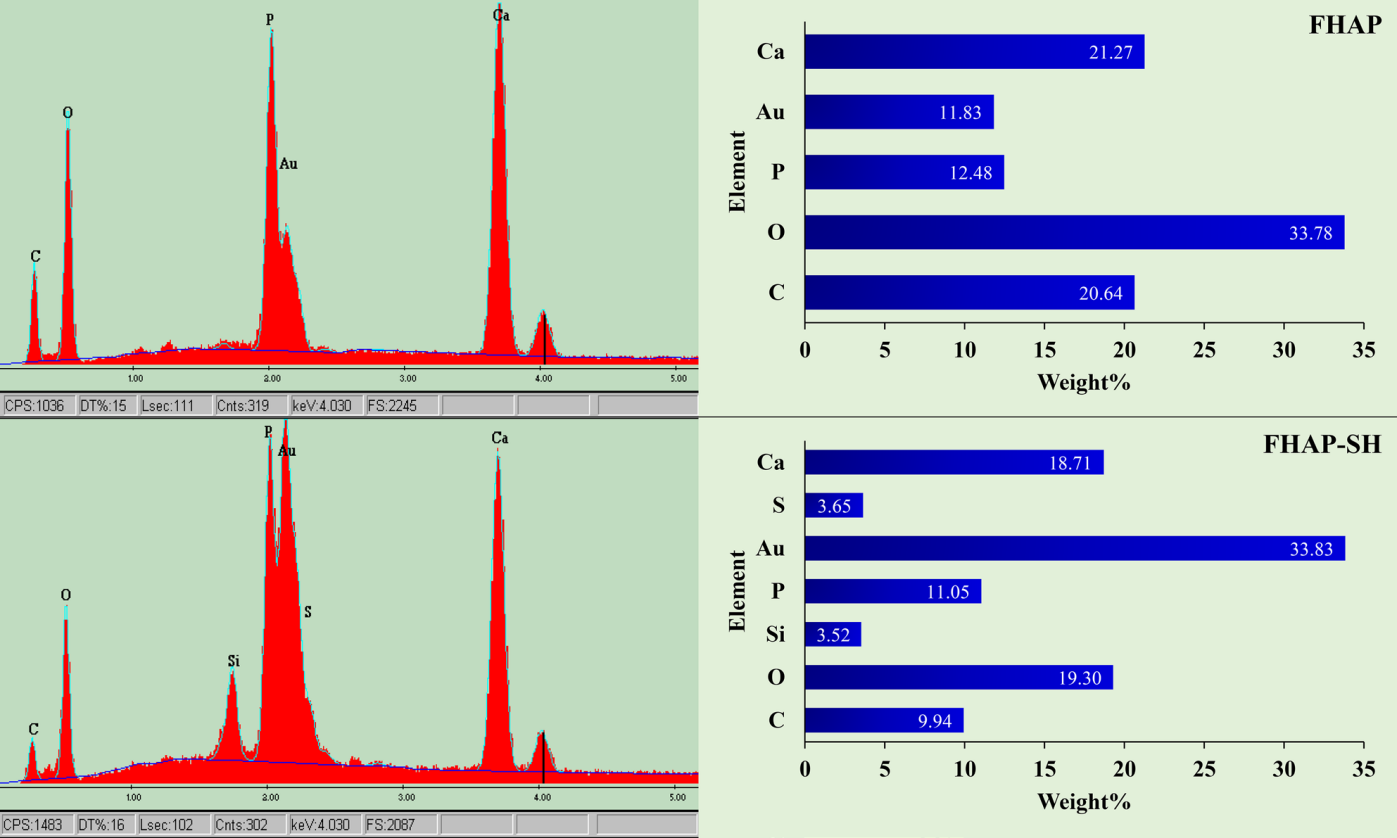
EDX spectra of the FHAP and FHAP-SH.

**Figure 4:**
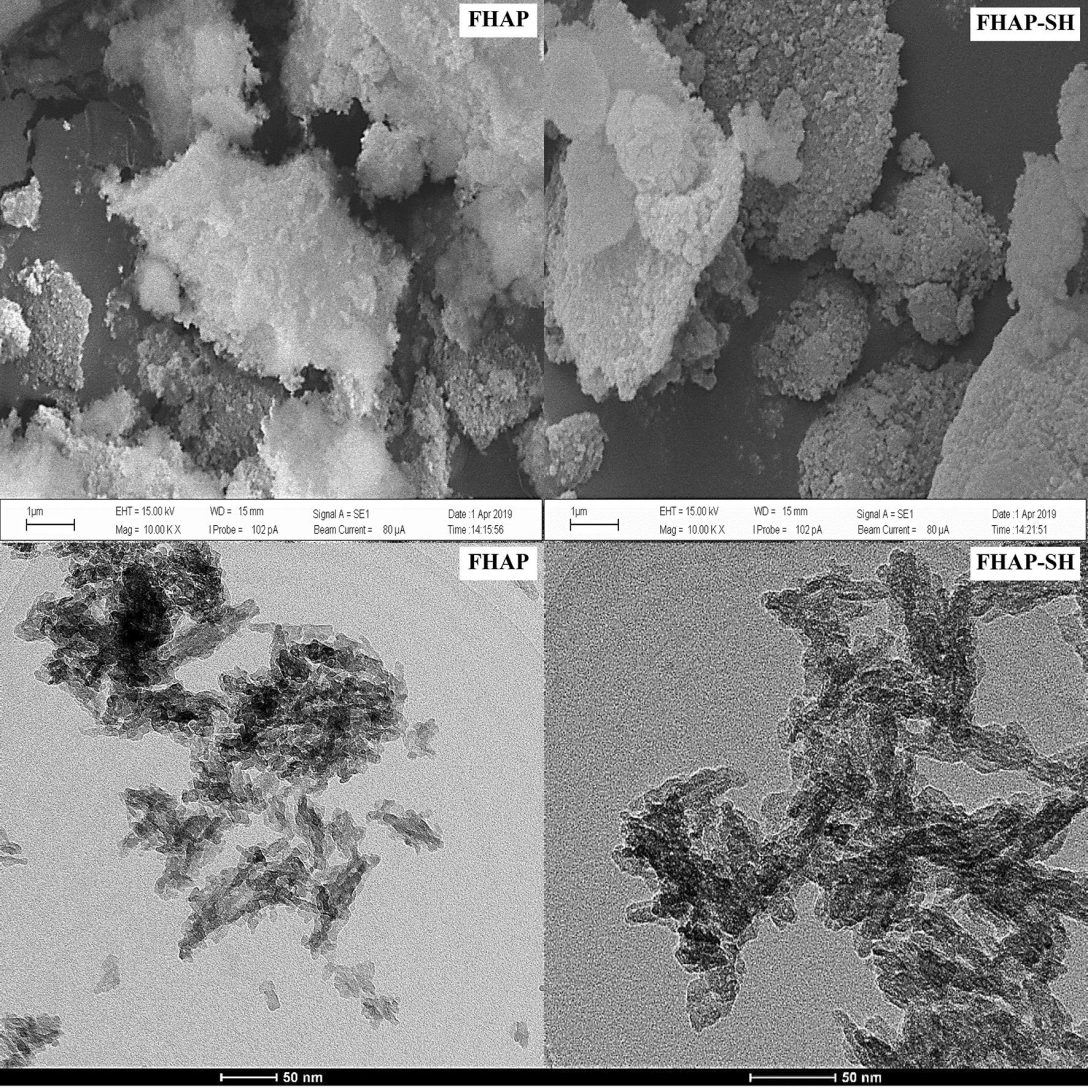
SEM and TEM images of the FHAP and FHAP-SH.

The XRD, FT-IR, EDX, SEM, and TEM of FHAP-SH results are shown in Figs. 1, 2, 3 and 4. As can be observed from the XRD pattern of FHAP-SH (Fig. 1), strong diffraction peaks of FHAP were present at 2*θ* = 25.9°, 31.7°, 40.1°, 46.8°, 49.6°, and 53.5°, which demonstrated the major pattern of FHAP^21^. The obtained diffraction peaks fitted with the standard JCPDS 00-009-0432 (hydroxyapatite) that has the (002), (102), (210), (211), (300), (202), (301), (310), (222), (213), and (004) planes^22,23^. In the thiol modification process, FHAP-SH was not changed in terms of the characteristic peak locations. However, the signal intensities were reasonably decreased as the crystal structure of the sorbent was covered by amorphous groups, which can be used to prepare the organosilane with a thiol group on the surface of the FHAP.

Figure 2 shows the FTIR spectra of FHAP and FHAP-SH. The 3400 cm^−1^ band corresponded to the hydroxyl (–OH) group stretching mode. The phosphate (PO_4_^3−^) vibrations of the prepared FHAP appeared at 1085, 1026, and 975 cm^−1^, while the bending vibrations of P–O–H for the inorganic Ca–P component appeared at 873 cm^−1^^24^. In the case of FHAP-SH, the stretching of the Si–O covalent linkages was verified by the presence of intense bands in the region between 1200 and 1100 cm^−1^ approximately, which indicates the formation of FHAP-SH. Evidence of some carbonate (CO_3_^2−^) substituents was found in both FHAP and FHAP-SH in the form of asymmetric stretching peaks located between 1581 and 1419 cm^−1^. These carbonates were produced under ambient CO_2_ dissolved during the FHAP-SH preparation step.

Furthermore, based on the EDX patterns shown in Fig. 3, both FHAP and FHAP-SH particles contained Ca, P, O, and C, evidencing the presence of hydroxyapatite. However, the Au spectrum also appeared because all the samples were coated with Au before observation under the electron microscope to improve the sample images. The changes in the spectral data of FHAP and FHAP-SH are also visibly displayed. The spectra of S and Si occurred only in FHAP-SH, which confirms the successful modification of hydroxyapatite with–SH. Therefore, hydroxyapatite now contained the attached–SH group, which could be used to remove the dyes from the samples.

The SEM and TEM images of FHAP and FHAP-SH are shown in Fig. 4, which display the needle-like or rice-like morphologies commonly reported by wet chemical precipitation methods^25^. The size of FHAP-SH was calculated to be 25.7 ± 4.9 nm in length and 7.5 ± 3.4 nm in diameter by using the ImageJ software version 1.53e (National Institutes Health, USA, https://imagej.nih.gov/ij). The nanocomposites exhibited a network of nanoparticle assemblages.

### Dye adsorption study

Different dye solutions (CR, CB, and MG) were prepared, and their removal efficiencies from aqueous media were evaluated for the synthesized FHAP-SH. The residual dye concentrations were analyzed by using a UV–vis spectrophotometer at 495, 595, and 620 nm for CR, CB, and MG, respectively. The equilibrium adsorption capacity (*q*_e_, mg g^−1^) was calculated as follows^26^:

1 $$q_{e} = \left( {C_{0} - C_{e} } \right) \times V/m$$

where *q*_e_ is the amount of the adsorbed organic dye at equilibrium (e.g., mg_Malachite Green_/g _adsorbent_),

*C*_0_ is the initial organic dye concentration in the solution (mg L^−1^), *C*_e_ is the equilibrium organic dye concentration in the solution after adsorption (mg L^−1^), *V* is the volume of the dye solution (L), and *W* is the weight of FHAP-SH (g).

### Effect of pH

**Figure 5:**
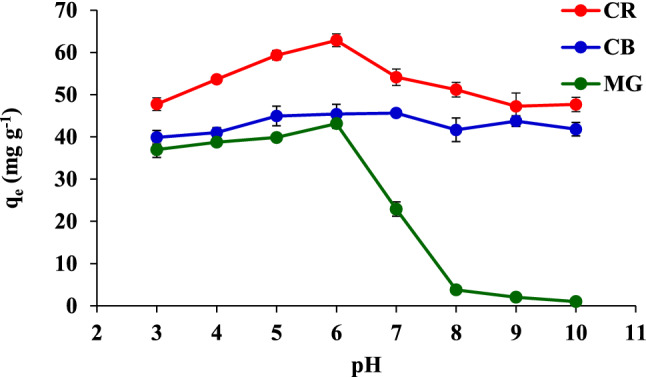
Effect of the pH value on the adsorption of CR, CB, and MG.

The pH value plays a critical role in adsorption. The pH (3–10) values of the adsorption of the three dyes presented in Fig. 5 show that the removal of CR and MG increased with an increase in the pH value from 3 to 6, with the best results obtained at pH 6. This result was attributed to the property of the CR dye, which is an anionic dye; however, the surface charge of the FHAP-SH is positive at pH 6, and the point of zero charge (pH_pzc_) of this FHAP-SH is at pH 7.73. Therefore, at pH lower < pH_pzc_, the FHAP-SH surface is positively charged, while at pH > pH_pzc_, the FHAP-SH surface is negatively charged. Therefore, the negatively cationic CR dyes can be easily adsorbed on the FHAP-SH surface. At a low pH of the sample, MG can also be significantly adsorbed on the FHAP-SH surface even though the charge of MG is positive. This is because the chemical interaction occurring between the MG dye and the FHAP may be related to the orientation property of the hydrogen bonds between the OH or SH group of the FHAP-SH and the amide group of MG. The UV–vis spectrometric analysis of MG revealed that the absorption at 620 nm was reduced at pH > 6. The color of the dye was self-fed. This may be due to the formation of a new species of the dye. In addition, the adsorption capacity decreased from pH 7 to 10, which may be due to the ionization constant (pK = 6.90) of the MG dye, which caused it to be 100% ionized at pH 4, 50% ionized at pH 6.9, 25% at 7.4 and 0% at pH 10.1^27^. In the case of CB, it was demonstrated to be existing in three forms depending on the pH of the solution: at pH < 0, the solution turns red (a cationic CB, absorbance peak at 470 nm); at pH = 1, the solution turns green (a neutral CB, absorbance peak at 650 nm); and at pH > 2, the solution turns blue (an anionic CB, absorbance peak at 595 nm). This means that at pH 6, the surface of FHAP-SH was protonated and positively charged. Consequently, an electrostatic interaction occurred between the FHAP-SH and CB (anionic dye). Overall, a minor difference in the signal of CB was observed at different pH values. Therefore, the pH value of 6 was preferred in subsequent experiments.

### Effect of the adsorbent dose

**Figure 6:**
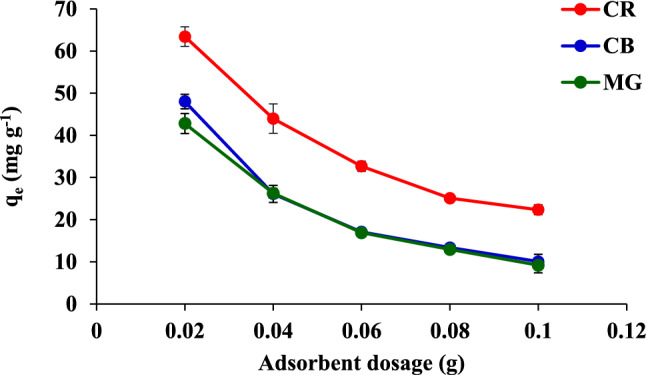
Effect of the adsorbent dose on the adsorption of CR, CB, and MG.

A relationship between *q*_e_ and the adsorbent dose is illustrated in Fig. 6. The adsorption capacities of CR, CB, and MG decreased rapidly with an increase in the adsorbent dose when the dose was higher than 0.02 g. This could be due to overlapping or aggregation at the adsorption positions in the overcrowding of FHAP-SH nanoparticles, which causes a decrease in the sorbents existing at the surface of these dyes. The highest *q*_e_ was obtained for CR, CB, and MG by using 0.02 g of the FHAP-SH powder. Therefore, subsequent experiments were conducted with an adsorbent dose of 0.02 g.

### Effect of ionic strength

**Figure 7:**
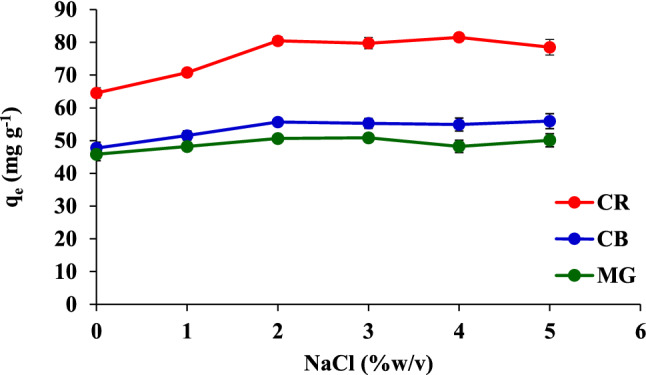
Effect of the ionic strength on the adsorption of CR, CB, and MG.

The ionic strength of the dye sample solutions was studied at pH 6 with 0.02 g of sorbent. An electrolyte concentration in a sample solution can be defined by its ionic strength, which is a property of the solution due to which the affinity between the solution and the sorbent phase gets influenced. The electrolyte addition would increase the aggregation of the dye molecules to promote the adsorption of the dyes and would decrease the solubility of the dyes^28^. As shown in Fig. 7, an increase in the concentration of NaCl increases the capacity of adsorption. In addition, the dye adsorptions did not change upon the addition of excess salt ions (> 2%) to the adsorption solution. Therefore, in the subsequent experiments, the ionic strength was set at 2% NaCl.

### Effect of ultrasonic power

**Figure 8:**
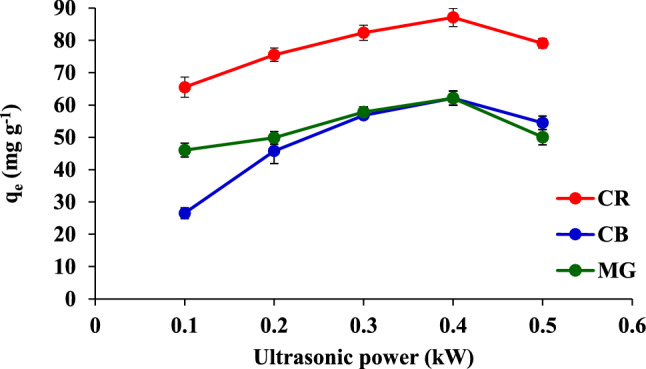
Effect of the ultrasonic power on the adsorption of CR, CB, and MG.

The adsorption process induced by the sonic wave is well known to accelerate the physical dispersion process^29^ due to the phenomenon of acoustic cavitation by a liquid, which leads to the mass transfer improvement in the system and thus increases the dye sorption strength. The study of the ultrasonic power revealed that *q*_e_ increases with an increase in the ultrasonic power from 0.1 to 0.4 kW (Fig. 8). Therefore, in the subsequent experiments, the ultrasonic power was set at 0.4 kW.

### Effect of temperature

**Figure 9:**
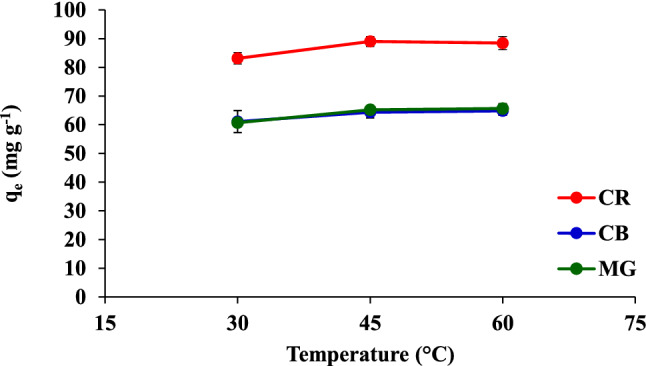
Effect of the temperature on the adsorption of CR, CB, and MG.

Figure 9 displays the adsorption capacities of CR, CB, and MG on the FHAP-SH at different temperatures. It was observed that *q*_e_ is slightly correlated with the temperature effect. The temperature of 45 °C presented the best relationship between the adsorption capacity and the adsorbents and was, therefore, used in the subsequent experiments.

### Effect of contact time

**Figure 10:**
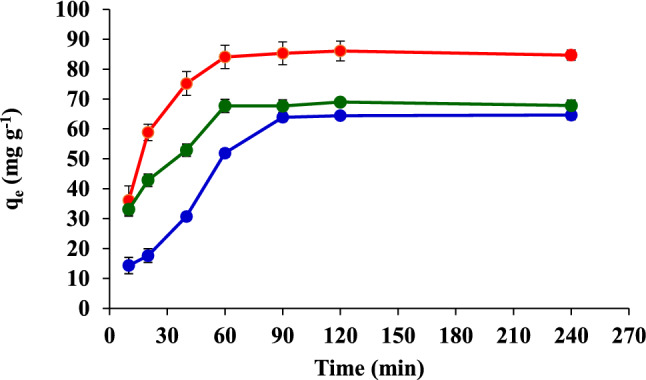
Effect of the contact time on the adsorption of CR, CB, and MG.

The effect of the contact time on CR, CB, and MG removal using FHAP-SH was studied at different contact times ranging between 10 and 240 min. It was observed that *q*_e_ increases sharply until the equilibrium is reached within 60 min for CR and MG and within 90 min for CB. In order to achieve maximum dye adsorption, the sonication times of 60, 90, and 60 min for CR, CB, and MG, respectively, were selected for subsequent experiments (Fig. 10).

### Adsorption isotherm

For the adsorption isotherm, the equilibria were fitted to the Langmuir and Freundlich isotherm models in a single dye system. For the Langmuir model, it was assumed that binding takes place at specific homogenous binding sites of the adsorbents, and it is a monolayer binding. The maximum adsorption occurs when the adsorbed molecules form a saturation layer on the adsorbent surface. The Langmuir equation of the isotherm can be written as follows^30^:

2 $$q_{{\text{e}}} = q_{\max } k_{l} c_{{\text{e}}} /1 + k_{{\text{l}}} c_{{\text{e}}}$$

where *q*_max_ is the maximum adsorption (mg g^−1^) and *k*_l_ is the Langmuir adsorption equilibrium constant (L mg^−1^).

The Freundlich adsorption isotherm is based on the concept of non-ideal adsorption on heterogeneous surfaces. The Freundlich model describes that the adsorption process on surface adsorption sites is exponentially distributed with respect to the heat of adsorption, and the stronger adsorption sites are saturated first, and the adsorption strength reduces with an increase in site occupation. It is expressed by the following relation^30^:

3 $$q_{{\text{e}}} = k_{{\text{f}}} \left( {c_{{\text{e}}} } \right)^{1/n}$$

where *k*_f_ is the Freundlich constant (mg^1−1/*n*^ L^1/*n*^ g^−1^), and *n* is the adsorption intensity. The fitted constants along with their regression coefficient *R*^2^ are shown in Table 1. The closer regression coefficient was in agreement with the best model fit. The Langmuir model was found to be reliable in describing the adsorption of CR, CB, and MG onto the FHAP-SH, with *R*^2^ values of ≥ 0.98. The impact of these dyes on the FHAP-SH surface was found to be more effective than the Freundlich model. The Langmuir isotherm displayed a better fit, which may suggest a homogenous distribution of active sites on the sorbent surface as the Langmuir model represents the local homogeneous surface. The maximum adsorption capacity (*q*_max_) of CR, CB, and MG was 500, 235, and 625 mg g^−1^, respectively. The *q*_max_ values of CR, CB, and MG removal by FHAP-SH were compared to those of the other previously reported adsorbents^31–41^. As shown in Table 2, the *q*_max_ values in this work were significantly higher than those previously reported for other adsorbents. In summary, the FHAP-SH nanoparticles could be used as adsorbent material for purifying water.

**Table 1:** The isotherm of CR, CB, and MG adsorptions on the FHAP-SH.

Dye	Langmuir model			Freundlich model		
	q_max_ (mg g^−1^)	K_L_ (L mg^−1^)	R^2^	n	K_F_ (L mg^−1^)	R^2^
CR	500	0.0071	0.9900	1.505	9.4864	0.9864
CB	235	0.0027	0.9885	1.828	18.668	0.9859
MG	625	0.0019	0.9969	1.349	9.9083	0.9890

**Table 2:** Adsorption capacity of CR, CB, and MG as compared to other adsorbents.

Adsorbent	dye	q_max_	Reference
Fe_2_O_3_@mSiO_2_	CR	88.29	31
Banana Peel Powder		164.6	32
HAP		305	33
CTAB modified CS beads		433.1	34
FHAP-SH		500	This work
α-Chitin Nanoparticles	CB	8.55	35
Starch/poly (alginic acid-cl-acrylamide)		31.24	36
Active carbon from *Ficus racemosa*		65.0	37
FHAP-SH		235	This work
Dead pine needles	MG	33.56	38
Activated carbon		50.34	39
Polyacrylic acid–nanoclay		243.11	40
Graphene Oxide		384.62	41
FHAP-SH		625	This work

### Adsorption thermodynamics

The adsorption thermodynamics parameters for CR, CB, and MG on FHAP-SH are presented in Table 3. At different temperatures of 303, 318, and 333 K, the thermodynamics parameters, such as Gibbs free energy change (Δ*G*°), enthalpy change (Δ*H*°), and entropy change (Δ*S*°), were studied. These parameters were calculated using the following expressions^30^:

4 $$\Delta G^\circ = {-}RT\ln K_{{\text{c}}}$$

5 $$K_{{\text{c}}} = C_{{\text{a}}} /C_{{\text{e}}}$$

6 $$\ln K_{{\text{c}}} = \Delta S^\circ /R - \Delta H^\circ /RT$$

**Table 3:** Adsorption thermodynamics of CR, CB, and MG adsorptions on the FHAP-SH.

Dye	ΔG° (kJ mol^−1^)			ΔH° (kJ mol^−1^)	ΔS° (J mol K^−1^)
	303 K	318 K	333 K		
CR	− 2598.78	− 3495.83	− 4361.36	0.0042	0.0320
CB	− 6634.77	− 7734.12	− 8992.87	17,155.94	78.44
MG	− 12,097.30	− 15,438.8	− 19,366.0	61,220.14	24.68

where *R* is the ideal gas constant (8.314 J mol^−1^ K^−1^), *T* is the adsorption temperature (K), and *K*_c_ is the adsorption equilibrium constant calculated as the ratio of the equilibrium concentration of the dye on the sorbent (*C*_a_) and the equilibrium concentration of the dye in the solution (*C*_e_). Δ*H*° and Δ*S*° were determined from the slope and the intercept of the plot of Δ*G*° versus *T*. The values of Δ*G*° (Table 3) were negative at all temperatures, indicating the feasibility of the process and the spontaneous nature of the adsorption of these dyes on FHAP-SH. An increase in the absolute Δ*G*° with an increase in the temperature suggests an augmented trend in the degree of the spontaneity of dye sorption at higher temperatures. In addition, positive Δ*H*° values confirm the endothermic adsorption reaction. The positive Δ*S*° values reflect the affinity of FHAP-SH toward these dyes and an increase in randomness at the solid-solution interface during adsorption.

### Adsorption kinetics

Adsorption kinetics can explain the solute uptake rate and the time requirement of the adsorption process. Sonication was performed at fixed time intervals from 10 to 240 min. The pseudo-first-order kinetics and the pseudo-second-order kinetics of the dye adsorption system could be calculated as follows^1^:

Pseudo-first order kinetics:

7 $$\log \left( {q_{{\text{e}}} - q_{{\text{t}}} } \right) = \log q_{{\text{e}}} - \left( {k_{1} /2.303} \right)t$$

Pseudo-second order kinetics:

8 $$t/q_{{\text{t}}} = 1/k_{2} qe_{2} - t/q_{{\text{e}}}$$

where *q*_e_ is the adsorption capacity at equilibrium (mg g^−1^), *q*_t_ is the amount adsorbed at a specific time (mg g^−1^), *t* is the time in hours (h), *k*_1_ is the rate constant of the pseudo-first-order kinetics (h^−1^), and *k*_2_ is the rate constant of the pseudo-second-order kinetics (g mg^−1^ h^−1^). The rate-determining processes for the CR, CB, and MG adsorptions are summarized in Table 4. Poor correlations were achieved for the linear form of the pseudo-first-order kinetic model (*R*^2^ = 0.2318–0.6660). The results indicate that the adsorption of these dyes onto FHAP-SH does not follow pseudo-first-order kinetics. However, the pseudo-second-order kinetics for the dyes adsorbed at the equilibrium were best fitted with the experimental adsorption data, with high correlations (*R*^2^ = 0.9945–0.9968). Therefore, the dye sorption on FHAP-SH could be approximated better by using the pseudo-second-order kinetic model rather than the first-order kinetic model.

**Table 4:** Adsorption kinetics of CR, CB, and MG adsorptions on the FHAP-SH.

Dye	q_e_, _exp_ (mg g^−1^)	Pseudo-first order kinetic model			Pseudo-second order kinetic model		
		q_e,cal_ (mg g^−1^)	k_1_ (h^−1^)	R^2^	q_e,cal_ (mg g^−1^)	k_2_ (g mg^−1^ h^−1^)	R^2^
CR	85.32	16.23	0.0033	0.6117	89.28	0.0014	0.9968
CB	62.89	28.18	0.0034	0.6660	77.52	0.0004	0.9945
MG	67.72	8.23	0.0026	0.2318	71.94	0.0014	0.9964

## Conclusion

A novel method of synthesizing 3-mercaptopropyl trimethoxysilane-modified hydroxyapatite derived from fish-scale residues by using ultrasound irradiation was discussed. The prepared materials were characterized by using XRD, FTIR, EDX, SEM, and TEM. Then, the adsorptions of CR, CB, and MG on the above-mentioned synthesized materials were performed and analyzed. Under optimized conditions, the three dyes CR, CB, and MG showed the maximum adsorption capacities of 500, 235, and 625 mg g^−1^, respectively, on FHAP-SH. The Langmuir model was selected to elucidate the adsorption behaviors with satisfactory correlation *R*^2^ values ranging from 0.9985 to 0.9969. The adsorption model followed an endothermic process for adsorption thermodynamics and the pseudo-second-order kinetic model for adsorption kinetics. This study presents an alternative environmental treatment and an efficient method for the removal of organic dyes from aqueous media.
